# *In Vitro* Drug Delivery of a Fixed-Dose Combination of Fluticasone Furoate/Umeclidinium/Vilanterol from a Dry Powder Inhaler

**DOI:** 10.1089/jamp.2021.0061

**Published:** 2023-02-09

**Authors:** Melanie Hamilton, Martin Anderson, Rajiv Dhand, Oonagh Patmore, David Prime, Edward Taylor

**Affiliations:** ^1^ GSK, R&D, Ware, Hertfordshire, United Kingdom.; ^2^ Karolinska Institutet, Stockholm, Sweden.; ^3^ Department of Medicine, Graduate School of Medicine, University of Tennessee Health Science Center, Knoxville, Tennessee, USA.

**Keywords:** asthma, chronic obstructive pulmonary disease, delivered dose, dose emission, peak inspiratory flow, peak inspiratory flow rate

## Abstract

**Background:**

Dry powder inhalers (DPIs) require patients to impart sufficient energy through inhalation to ensure adequate dose emission, medication deaggregation, and resultant particle sizes suitable for lung deposition. There is an ongoing debate regarding the level of inspiratory effort, and therefore inspiratory flow rate, needed for optimal dose delivery from DPIs.

**Materials and Methods:**

The delivered dose (DD) and fine particle fraction (FPF) for each component of fluticasone furoate/umeclidinium/vilanterol (FF/UMEC/VI) 100/62.5/25 μg and FF/UMEC/VI 200/62.5/25 μg ELLIPTA DPIs were assessed at flow rates of 30, 60, and 90 L/min. Electronic lung (eLung) (eLung; an electronic breathing simulator) assessments were conducted to replicate inhalation profiles representing a wide range of inhalation parameters and inhaled volumes achieved by patients with chronic obstructive pulmonary disease (COPD) or asthma of all severity levels. Timing and duration of dose emission were assessed using a particle detector located at the entrance of an anatomical throat cast attached to the eLung.

**Results:**

During DD assessment, a mean of >80% of the nominal blister content (nbc) was emitted from the ELLIPTA DPI at all flow rates. In Next Generation Impactor assessments, the observed mean DD across flow rates for FF/UMEC/VI 100/62.5/25 μg ranged from 85.9% to 97.0% of nbc and 84.0% to 93.5% for FF/UMEC/VI 200/62.5/25 μg. In eLung assessments, 82.8% to 95.5% of nbc was delivered across the PIF range, 43.5 to 129.9 L/min (COPD), and 85.1% to 92.3% across the PIF range, 67.4 to 129.9 L/min (asthma). The FPF (mass <5 μm; % nbc) for each component was comparable across all flow rates and inhalation profiles. Dose emission timings indicated that near-complete dose emission occurs before reaching PIF.

**Conclusions:**

Dose delivery assessments across all flow rates and inhalation profiles indicate that patients with all severity levels of COPD or asthma can achieve the required inspiratory effort for efficient delivery of all components of FF/UMEC/VI from the ELLIPTA DPI. Dose emission profiles suggest rapid and near-complete dose delivery from the ELLIPTA DPI before reaching PIF.

## Introduction

Inhaled therapies are the cornerstone of pharmacological management of chronic obstructive pulmonary disease (COPD) and asthma.^1,2^ Inhalation devices encompass a wide variety of delivery systems, including pressure-driven sprays (pressurized metered-dose inhalers [pMDIs] and soft mist inhalers), air-driven nebulizers, and dry powder inhalers (DPIs).^3^ As a class, DPIs vary in design (e.g., swirl chambers, mouthpieces), which results in varying levels of inspiratory resistance and differing flow rate characteristics.^4^

Besides device-specific properties and the patient's disease state, the anatomical structure of the mouth and throat and inspiratory volume are two other important patient-related factors that can impact aerosol delivery to the lung.^3^ Additionally, pMDIs require patients to successfully coordinate dose actuation and their inhalation to ensure optimal delivery of medication to the lungs, while DPIs require patients to achieve sufficient inspiratory effort and therefore inspiratory flow rate.^3^

The deposition pattern in the respiratory tract is highly dependent on particle size.^3^ Large particles have a high probability to be deposited in the upper throat (oropharynx), while small particles have a high probability to reach the lower airways and alveolar compartment.^3^ The particles within an aerosol that are <5 μm in size are termed fine particles.^3^

The ELLIPTA DPI is a moderate-resistance, single-step activation multidose inhaler supplied in either a single-strip (monotherapy) or two-strip (combination therapy) configuration for patients with COPD or asthma.^5^ For patients with COPD, the ELLIPTA DPI delivers umeclidinium (UMEC) monotherapy, UMEC/vilanterol (UMEC/VI) dual therapy, fluticasone furoate/VI (FF/VI) dual therapy, and FF/UMEC/VI triple therapy. For patients with asthma, it delivers FF monotherapy, FF/VI dual therapy, and FF/UMEC/VI triple therapy.^6–9^ Currently, FF/UMEC/VI 100/62.5/25 μg is approved for patients with COPD or asthma, while FF/UMEC/VI 200/62.5/25 μg is only approved for patients with asthma.^7^ The product strength for FF/UMEC/VI is also referred to as the nominal blister content (nbc).^7,10^

Both the Global Initiative for Chronic Obstructive Lung Disease (GOLD) and Global Initiative for Asthma management strategies stress the importance of inhaler technique training to improve symptom control and recommend that patients who cannot master a device should switch inhalers.^1,2^

Studies have shown that DPIs are frequently preferred over pMDIs by patients with COPD or asthma,^11^ with the ELLIPTA DPI being associated with fewer errors in use, greater patient preference, and more patients rating it as easy to use compared with other inhalers.^12,13^ Furthermore, DPIs have been shown to have an annual carbon footprint 20–30 times smaller than pMDIs, indicating that DPIs also offer greater environmental benefits.^14^

Concerns have been raised that with DPIs, dose delivery and subsequent medication deposition in the airways may be inadequate in patients with low peak inspiratory flow (PIF) values.^15–17^ However, *in vitro* studies have demonstrated consistent dose delivery of the components of FF/UMEC/VI ELLIPTA DPI at flow rates ranging from 30 to 90 L/min under standard test conditions and 43.5 to 129.9 L/min using the electronic lung (eLung) breathing simulator developed for characterization of inhalation devices through replication of patient-specific inhalation profiles.^5,18^

Here we report the *in vitro* dosing performance of the FF/UMEC/VI ELLIPTA DPI when characterized using a routine methodology (i.e., delivered dose (DD) and cascade impaction testing) and by eLung assessments. We also report the relationship between timing and duration of dose emission from the ELLIPTA DPI and the inspiratory flow rate to identify when, in the inhalation profile, the medication is released and therefore the importance of PIF and inhaled volume on dosing.

## Materials and Methods

All data in this article are *in vitro* analyses and therefore informed consent, ethics committee or institutional review board (IRB) approval were not required.

**FIG. 1.**
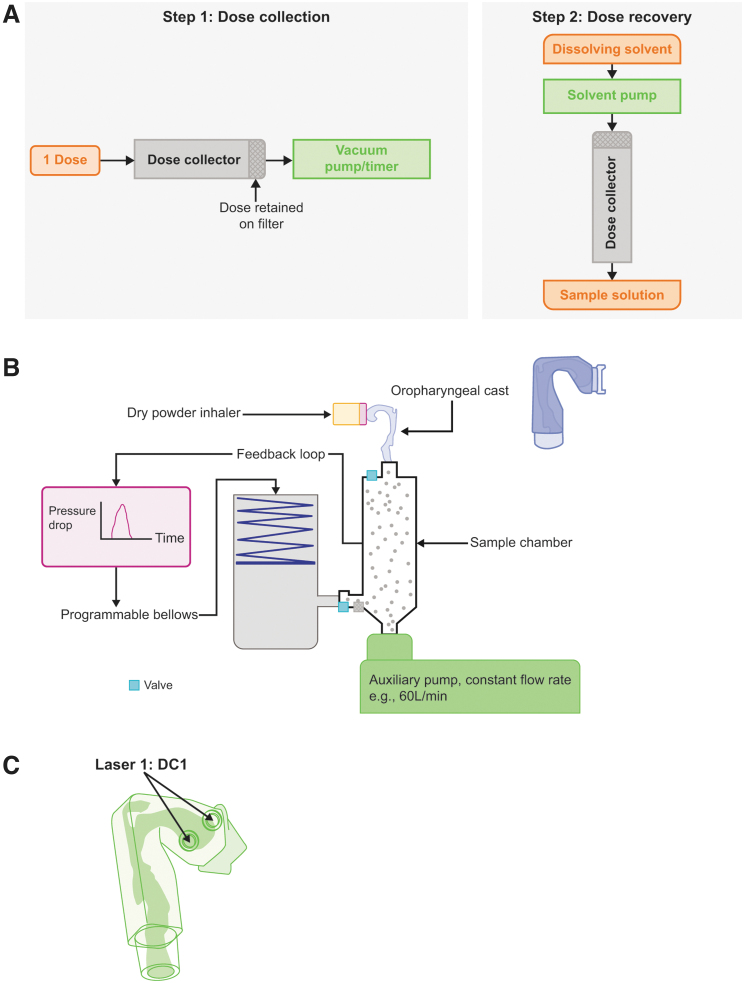
Experimental setups for **(A)** DD assessments; **(B)** eLung assessments; and **(C)** laser position for particle detector assessments. DD, delivered dose; eLung, electronic lung.

Detailed methodologies for DD, Next Generation Impactor (NGI), and eLung assessments have been provided previously,^5,18^ and details of the experimental setups are provided in Figure 1. Testing was performed for all components (FF, UMEC, and VI) of a single batch of the FF/UMEC/VI ELLIPTA DPI at each product strength: 100/62.5/25 μg and 200/62.5/25 μg.^7,10^

### DD measurements

The total dose delivered from the ELLIPTA DPI was assessed at flow rates of 30, 60, and 90 L/min. These flow rates incorporate and extend beyond the lowest PIF (43.5 L/min) achieved by patients with all severity levels of asthma or COPD by inhaling through the ELLIPTA DPI (two-strip configuration) in the RES113817 (NCT01345266) and RES117178 (NCT02076269) studies.^19^

At each flow rate, two doses from each of 10 inhalers were aerosolized into the test apparatus. To assess the total amount of dose delivered, the dose collector was rinsed with a suitable solvent to recover the deposited mass, and solutions were analyzed using high-performance liquid chromatography (HPLC) (HPLC conditions are provided in the Supplementary Data).

### Aerodynamic particle size distribution by NGI

The aerodynamic particle size distribution (APSD) of each component of FF/UMEC/VI was determined using an NGI (NGI MSP Corporation Model M170, Preseparator, USP Induction Port with GSK integrated mouthpiece)^19–21^ at flow rates of 30, 60, and 90 L/min with an inhaled volume of 4 L.

For each product strength and flow rate, four ELLIPTA DPIs were tested, with six doses actuated into the impactor from each device, resulting in a six-dose composite sample for analysis. The induction port and all stages of the NGI apparatus were rinsed with a suitable solvent to recover the deposited mass, and solutions were analyzed using HPLC (see Supplementary Data for details).

Total DD and fine particle fraction (FPF) (mass <5 μm; % nbc) were subsequently determined from the results obtained.

### eLung measurements

The eLung apparatus uses an anatomical throat cast (GSK) considered to be more structurally representative of a human throat than the metal throat used in standard *in vitro* inhaler testing. Although the oropharyngeal anatomical structure varies in the general population, the cross-sectional area of the eLung throat cast closely matches those observed in imaging studies of healthy individuals as well as patients with asthma and COPD and therefore was suitable for use in these experiments.^18,22^

Maximal effort, inhaler-specific inhalation profiles were previously recorded from patients with mild-to-very severe COPD (GOLD stage I–IV) or mild-to-severe asthma (British Thoracic Society steps 1–5) in the RES113817 and RES117178 studies. Patient demographics have been described previously.^19^

Five inhalation profiles representing populations with COPD and asthma of all severity levels were selected from these studies and replicated using the eLung. These inhalation profiles were selected to include the absolute minimum (43.5 L/min) and maximum (129.9 L/min) PIFs observed across the patient populations for which the FF/UMEC/VI ELLIPTA DPI is approved (COPD and asthma) and the median and interquartile range PIFs. Specifically, profiles with nominal PIFs of 67.4, 82.4, 99.9, and 129.9 L/min were used to represent both COPD and asthma populations, in addition to 43.5 L/min for COPD and 113.1 L/min for asthma.

To allow characterization of the APSD of the dose passing beyond the anatomical throat, an NGI was attached to the eLung and operated at a standard flow rate of 60 L/min after replication of the patient-specific inhalation profile was completed. As per the NGI analysis described above, six doses were actuated from each of the three devices to form a six-dose composite sample for analysis.

To assess the total DD and FPD, individual parts of the eLung/impactor apparatus were rinsed with a suitable solvent to recover the deposited mass and solutions analyzed using HPLC (see Supplementary Data for details). Each test was performed in triplicate for each inhalation profile.

### Particle detector measurements in conjunction with the eLung

The dose emission timing profile was assessed using a light-emitting diode (635 nM, 1 mW) and a photodiode and transimpedance amplifier supplied by Si-Plan electronics using a process similar to that described by Ziffels et al.^23^ The equipment was aligned at the entrance to the anatomical throat attached to the eLung (Fig. 1C), which provides the synchronization signal at the start of the inhalation replication.

Three inhalation profiles with PIFs of 30, 43.5, and 129.9 L/min were replicated through the eLung using this setup, the dose emission profile was recorded, and the mean of six doses was plotted relative to the inhalation profile. The inhalation profile with PIF of 30 L/min was previously recorded from a standard NGI setup using an inhalation profile recorder (GSK) and replicated using the eLung in the same way as the patient-specific profiles with PIFs of 43.5 and 129.9 L/min.

### Statistical analysis

The total DD and FPF for all assessments were calculated as mean percentage (range) of the nbc of each component. For each response, data were analyzed using a two-way ANOVA approach with fixed factors of product (levels: 100/62.5/25 μg and 200/62.5/25 μg) and flow rate (levels: 30, 60, and 90 L/min for NGI/DD; and minimum, 25%; medium, 75%; and maximum for eLung).

For each combination of product and flow rate, the predicted mean from the analysis and tolerance interval (95%, 95%) were calculated. This tolerance interval corresponds to the range (with 95% confidence) in which 95% of all future doses of FF/UMEC/VI should fall.

## Results

### DD assessment

**FIG. 2.**
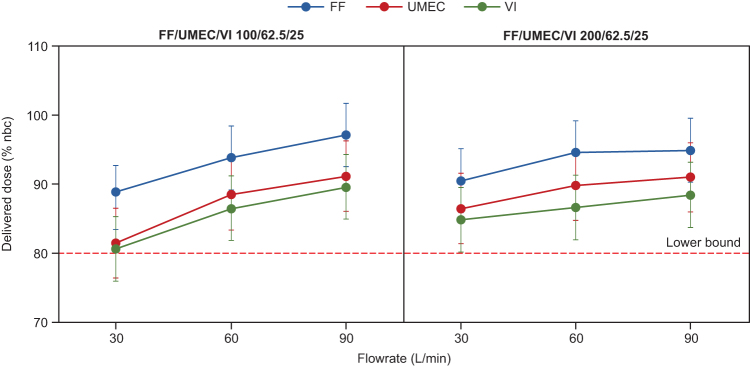
Mean DD of FF/UMEC/VI through the ELLIPTA DPI (percentage of nominal blister content) for FF/UMEC/VI 100/62.5/25 μg and FF/UMEC/VI 200/62.5/25 μg (DD assessment). Dose delivery performance of the ELLIPTA DPI was assessed at flow rates of 30, 60, and 90 L/min (four replicates at each flow rate). Error bars indicate 95% confidence and 95% coverage tolerance intervals around the mean. DPI, dry powder inhaler; FF, fluticasone furoate; nbc, nominal blister content; UMEC, umeclidinium; VI, vilanterol.

Mean DDs for FF, UMEC, and VI were >80% of the nbc for all flow rates and both strengths of the triple combination ELLIPTA DPI (Fig. 2; Supplementary Table S1). For FF/UMEC/VI 100/62.5/25 μg, DD tolerance intervals ranged between 83.3% and 101.8% for FF, 76.3% and 96.3% for UMEC, and 75.8% and 94.3% for VI across all three flow rates. For FF/UMEC/VI 200/62.5/25 μg, DD tolerance intervals ranged from 85.8% to 99.6%, 81.3% to 96.1%, and 80.1% to 93.2% of the nbc across all three flow rates for FF, UMEC, and VI, respectively.

### APSD by NGI

**FIG. 3.**
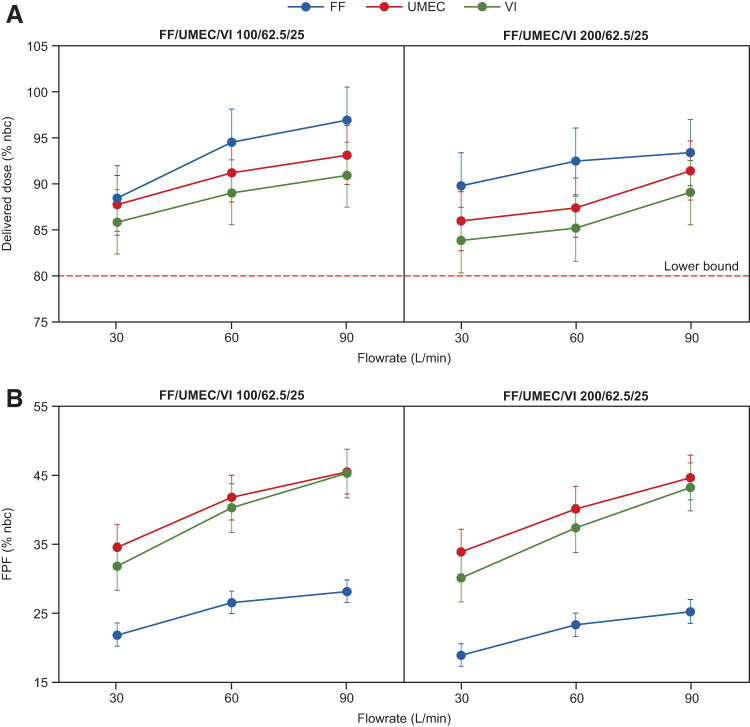
Mean total DD **(A)** and FPF **(B)** of FF/UMEC/VI through the ELLIPTA DPI as measured through an NGI (percentage of nominal blister content) for FF/UMEC/VI 100/62.5/25 μg and FF/UMEC/VI 200/62.5/25 μg (cascade impaction assessment). Dose delivery performance of the ELLIPTA DPI was assessed by the NGI at flow rates of 30, 60, and 90 L/min with an inhaled volume of 4 L and four replicates at each flow rate. Error bars indicate 95% confidence and 95% coverage tolerance intervals around the mean. FPF, fine particle fraction; NGI, Next Generation Impactor.

For FF/UMEC/VI 100/62.5/25 μg, DD tolerance intervals ranged from 84.9% to 100.6% of the nbc for FF, 84.5% to 96.5% for UMEC, and 82.4% to 94.6% for VI across all three flow rates (Fig. 3A; Supplementary Table S2). For FF/UMEC/VI 200/62.5/25 μg, mean DD tolerance intervals ranged from 86.3% to 97.1%, 82.8% to 94.8%, and 80.4% to 92.7% of the nbc for FF, UMEC, and VI, respectively (Fig. 3A).

FPF tolerance intervals for FF/UMEC/VI 100/62.5/25 μg ranged from 20.2% to 29.9% of the nbc for FF, 31.3% to 48.8% for UMEC, and 28.3% to 48.8% for VI (Fig. 3B). For FF/UMEC/VI 200/62.5/25 μg, FPF tolerance intervals ranged from 17.3% to 27.1%, 30.7% to 48.0%, and 26.7% to 46.9% of the nbc for FF, UMEC, and VI, respectively (Fig. 3B; Supplementary Table S2).

The median mass aerodynamic diameter (MMAD) decreased with increasing flow rates (Supplementary Table S2).

### eLung measurements

**FIG. 4.**
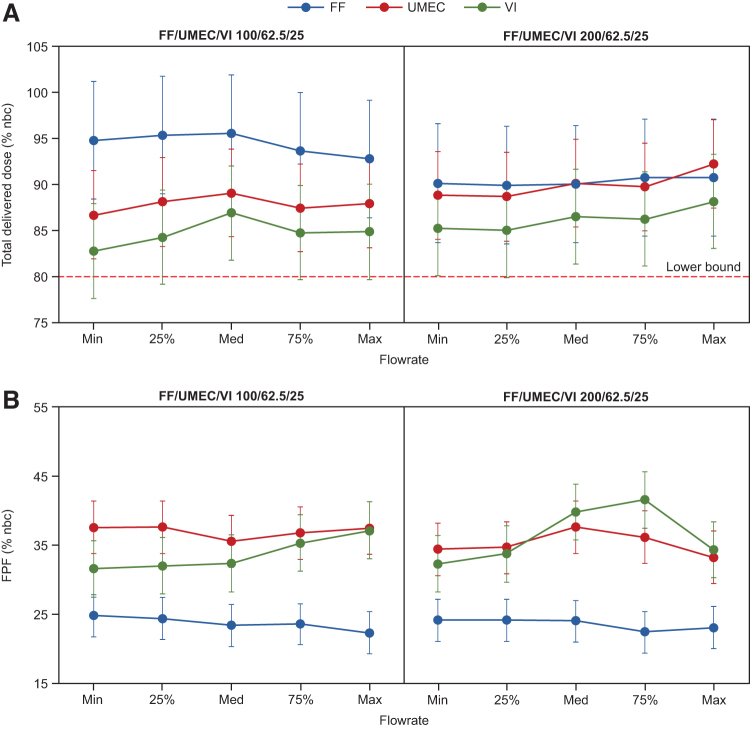
Mean total DD **(A)** and FPF **(B)** of FF/UMEC/VI through the ELLIPTA DPI as measured using the eLung* for FF/UMEC/VI 100/62.5/25 μg and FF/UMEC/VI 200/62.5/25 μg. *Dose delivery performance of the ELLIPTA DPI was assessed using the eLung to replicate inhalation profiles previously recorded from patients with COPD or asthma (RES113817 and RES117178 studies) with three replicates for each flow rate. Error bars indicate 95% confidence and 95% coverage tolerance intervals around the mean. COPD, chronic obstructive pulmonary disease.

For FF/UMEC/VI 100/62.5/25 μg and using inhalation profiles representing COPD and asthma populations with PIF ranging from 43.5 to 129.9 L/min, DD tolerance intervals ranged from 86.4% to 102.0% of the nbc for FF, 81.9% to 93.9% for UMEC, and 77.6% to 92.1% for VI (Fig. 4A; Supplementary Table S3). FPF tolerance intervals ranged from 19.2% to 27.9%, 31.7% to 41.4%, and 27.4% to 41.3% for FF, UMEC, and VI, respectively (Fig. 4B).

For FF/UMEC/VI 200/62.5/25 μg and using inhalation profiles representing an asthma population with PIF ranging from 67.4 to 129.9 L/min, DD tolerance intervals ranged from 83.6% to 97.2% of the nbc for FF, 83.9% to 97.1% for UMEC, and 79.9% to 93.4% for VI (Fig. 4A; Supplementary Table S3). FPF tolerance intervals ranged from 19.4% to 27.3%, 29.4% to 41.5%, and 28.2% to 45.7% for FF, UMEC, and VI, respectively (Fig. 4B). The MMAD decreased with increasing flow rates (Supplementary Table S3).

### eLung and particle detector measurements

**FIG. 5.**
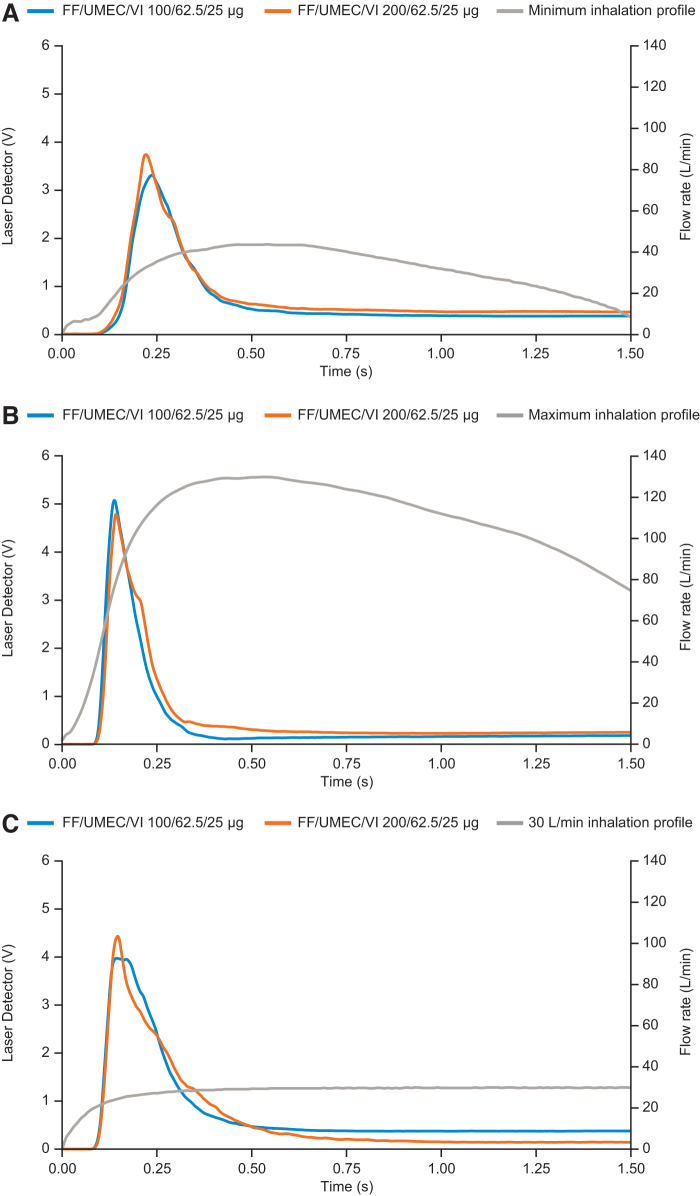
Time and duration of dose emission relative to inhalation profiles. Dose emission generated by **(A)** minimum inhalation profile of PIF at 43.5 L/min; **(B)** maximum inhalation profile of PIF at 129.9 L/min; and **(C)** inhalation profile of 30 L/min. PIF, peak inspiratory flow.

For all three profiles (PIFs of 30, 43.5, and 129.9 L/min), dose emission was observed to begin within 0.1 seconds of the start of inhalation and was largely complete by 0.5 seconds, before PIF is reached (Fig. 5).

## Discussion

The mean DD results obtained in three separate assessments (DD, NGI, and eLung) in conjunction with the dose emission profiles recorded using a particle detector demonstrate that delivery of all active components within the FF/UMEC/VI ELLIPTA DPI is >80% of the nbc across a wide range of inspiratory flow rates that are representative of those achieved by patients with COPD or asthma of all severity levels. The lower bounds of the tolerance intervals indicate that the majority of all future doses of all components are predicted to be >75% of the nbc.

For all components of the FF/UMEC/VI ELLIPTA DPI across flow rates ranging from 30 to 90 L/min, over 80% of the nbc was delivered from the inhaler in the DD assessment, while over 84% of the nbc was recorded in the NGI analysis over the same flow rate range. By considering tolerance intervals, the majority of future doses are also predicted to be >75% in the DD assessment and 80% in the NGI assessment.

These results confirm that an adequate quantity of the blister content of FF/UMEC/VI is delivered at all flow rates tested under standardized *in vitro* test conditions and that delivery performance is not compromised at the lowest flow rate of 30 L/min, which is below the minimum detected flow rate of 43.5 L/min recorded in the RES113817 and RES117178 studies.^19^

These data are supported by those of the eLung assessments, which demonstrated comparable (i.e., 95%, 95% tolerance intervals overlapped) total DD and FPF across the wide range of inhalation parameters assessed, including a PIF range of 43.5 to 129.9 L/min and inhaled volume of 0.8 to 3.2 L. The eLung data indicate largely flow-independent delivery of FF, UMEC, and VI through the ELLIPTA DPI across both the 100/62.5/25 μg and 200/62.5/25 μg product strengths.

The eLung has been designed to be used with an anatomically representative oropharyngeal cast, allowing for a more realistic assessment of dose delivery to the airways than standard *in vitro* testing with an NGI and metal induction port.^18^ Thus, by replicating inhalation profiles recorded from patients in the RES113817 and RES117178 studies,^18,19^ these eLung assessments can also provide evidence for the likely efficiency of dose delivery through the ELLIPTA DPI in patients with all severity levels of COPD or asthma.

In the RES113817 and RES117178 studies, the lowest recorded PIF with a two-strip ELLIPTA DPI configuration was 43.5 L/min in a patient with very severe COPD and 67.4 L/min in a patient with moderate asthma (of note, the lowest recorded PIF in patients with severe asthma was 72.4 L/min).^19^ The inhalation profiles representing the minimum PIF recorded from each patient population (COPD and asthma) and replicated in eLung experiments resulted in a mean DD of at least 82.7% and 85.3% of the nbc, respectively, across all components.

The tolerance interval for the lowest PIF revealed that all future doses are predicted to be ≥78% of the nbc. Furthermore, disease severity in asthma and COPD is assessed through the exhalation portion of the flow–volume curve (i.e., FEV_1_ and FVC).^1,2^ The high percentage of DD in this analysis indicates that disease severity is not the most important parameter for assessing inhaler suitability as even patients with very severe COPD or severe asthma have enough inspiratory effort to achieve PIF through the ELLIPTA DPI, which results in efficient dose delivery of all components of FF/UMEC/VI.

Use of DPIs requires production of fine particles (aerodynamic diameter <5 μm) through deagglomeration of the formulation, which for some DPIs may improve with increasing energy.^3^ Thus, it is of interest to consider the proportion of FF/UMEC/VI delivered as fine particles across a wide range of inhalation parameters that are representative of the intended patient population.

Overall, the tolerance intervals revealed that the FPF recorded as a percentage of the nbc in the NGI analysis for both treatment strengths is expected to range from 17.3% to 29.9% for FF, 30.7% to 48.8% for UMEC, and 26.7% to 48.8% for VI across the three flow rates assessed. Data from eLung assessments further demonstrated deagglomeration as the FPF was similar for each component across PIFs ranging from 43.5 to 129.9 L/min, suggesting that patients with all severity levels of COPD and asthma can achieve an optimal flow rate through the ELLIPTA inhaler.

Additionally, the MMAD was shown to decrease and FPF increase with greater flow rates, irrespective of which method was used. The association between the FPF and clinical efficacy is a complex issue and is dependent on a number of factors, including formulation type and differences in physiochemical characteristics between drugs, with inconsistent results reported across the literature.^24^ However, the FPF reported in these analyses is consistent with those seen for other DPIs^25^; furthermore, the consistency of aerosolized performance across the wide range of inhalation parameters tested makes the ELLIPTA DPI suitable for patients with all severity levels of COPD or asthma.

Additionally, the ELLIPTA inhaler contains the drug powder in blisters, rather than as compressed powder in one large compartment, and this may contribute to the efficiency of dose delivery with the ELLIPTA DPI. The testing methodologies used in this study employ different mechanisms regarding filtration of the aerosol cloud and flow rate setup, which likely explain any differences in trends within the results in our study.

The timing of dose emission indicates that regardless of PIF, emission from the ELLIPTA DPI starts within 0.1 seconds and most of the dose is emitted within 0.5 seconds, suggesting that dose delivery occurs at very low flow rates and inhaled volumes. This timing of emission is due to the design of the ELLIPTA DPI, which has a short airflow channel that may reduce the dose lost to internal surfaces, thereby helping maintain the percentage of dose emitted as well as flow properties of the product formulation.^5^

Furthermore, for each inhalation profile, PIF occurred after the majority of dose emission had taken place, suggesting that dose emission is not fully dependent on PIF. However, it should be noted that PIF has shown a relationship with other inhalation parameters, such as pressure slope and acceleration rate, both of which impact the delivery of medication from a DPI.^26^

In a recent *post hoc* analysis on two clinical trial populations (207608/207609) and a real-world database (Kaiser Permanente Northwest) population, nearly all (>99%) patients with COPD could achieve spirometric PIF values that were estimated to be equivalent to a PIF of ≥30 L/min through the ELLIPTA DPI.^27^ This finding is supported by a recent real-world study in patients with self-reported COPD or asthma, which demonstrated that 100% of patients achieved a baseline PIF of ≥30 L/min, as measured using the In-Check DIAL (Clement Clarke International; Harlow, UK) at a low–medium DPI resistance setting.^28^

Furthermore, a lack of correlation between spirometric PIF at screening and clinical outcomes in patients with moderate-to-severe COPD was also seen in the 207608/207609 clinical trial populations, which aligns with the flow-independent and consistent dose delivery from the ELLIPTA DPI across a wide range of PIFs achieved by patients with COPD of all severity levels.^27^ This is further supported by the efficacy seen with FF/UMEC/VI in large-scale Phase 3 trials in patients with inadequately controlled asthma and moderate-to-severe COPD.^29–31^

The dose delivery with the ELLIPTA DPI reported in these assessments compares favorably with that reported for other DPIs. In an *in vitro* study assessing dose delivery across seven inhalation profiles, the Breezhaler DPI reported a mean DD of 68% of the labeled dose of indacaterol, 150 μg, and the HandiHaler DPI reported a mean DD of 42% of the labeled dose of tiotropium, 18 μg.^32^

In addition, for the Breezhaler and HandiHaler DPIs, the mean FPF (defined as ≤4.7 μm) delivered was 26.8% of the labeled dose of indacaterol and 10.0% of the labeled dose of tiotropium 18 g respectively.^32^ The lowest recorded PIFs through the Breezhaler and HandiHaler were 47 and 23 L/min, respectively, in this study, which resulted in DDs of 55% and 37% and FPFs of 18% and 8% of the labeled dose, respectively.^32^

A limitation of this study is that the eLung assessments used the range of inhalation profiles obtained in the RES113817 and RES117178 studies, which included a relatively small number of patients (*n* = 120), and thus a larger population size may have extended the range of PIFs observed. However, it should be noted that patients with PIF values below the minimum recorded in the RES113817 and RES117178 studies are rarely seen and inclusion of DD and NGI assessments enabled investigation of a flow rate (30 L/min) below this minimum observed PIF, supporting the performance of the ELLIPTA DPI at low PIF values.

Furthermore, the use of the eLung allowed replication of patient-specific inhalation profiles, which not only incorporated a wide range of PIF values but also a range of other inhalation parameters, including inhalation volumes and flow acceleration characteristics.^19^ An additional point to note is that the duration of flow rate measurement was consistent across all flow rates for the DD test. Therefore, the volume of inhalation did not remain consistent across all flow rates for the dose delivery data. However, the results presented are still robust as the dose is delivered within a short period of time and thus the volume would have had a negligible effect on the dose and delivery obtained.

## Conclusions

These *in vitro* analyses demonstrate efficient delivery of each component of FF/UMEC/VI in the ELLIPTA DPI at flow rates as low as 30 L/min and across a wide range of inhalation parameters representative of patients with all severity levels of COPD and asthma. Additionally, the dose emission profiles suggest that rapid dose delivery from the ELLIPTA DPI is achieved, with the majority of the dose delivered before PIF is reached.

These data show that patients with COPD or asthma of all severity levels can achieve inspiratory flow profiles that result in efficient dose delivery of FF/UMEC/VI through the ELLIPTA DPI.

## Supplementary Material

Supplemental data

## Data Availability

Anonymized individual participant data and study documents can be requested for further research from www.clinicalstudydatarequest.com

## Notes

Reviewed by:

Guenther Hochhaus Michael Hindle
