# Supplementary material for: In Vitro Drug Delivery of a Fixed-Dose Combination of Fluticasone Furoate/Umeclidinium/Vilanterol from a Dry Powder Inhaler
Source: J Aerosol Med Pulm Drug Deliv. 2023 Feb 9;36(1):34–43. doi: 10.1089/jamp.2021.0061 (PMC9942181; doi:10.1089/jamp.2021.0061)
Supplement: Supplemental data [file Supp_Data.docx]

**Supplementary material**

**Supplementary methods**

**HPLC conditions:** An Agilent Zorbax SB-C18 1.8 micron, 5 cm x 4.6 mm column was used. The mobile phase consisted of: (A) Water acidified with 0.1% trifluoroacetic acid (TFA) and (B) acetonitrile (ACN) acidified with 0.1% TFA. The following gradient was used:

| **Time (mins)** | **% Mobile Phase A** | **% Mobile Phase B** |
| --- | --- | --- |
| 0.00  1.50  1.51  2.50 | 70  20  70  70 | 30  80  30  30 |

The HLPC was carried out at a temperature of 40°C, with a flow rate of 2 mL/min, and an injection volume of 20 µL. UV detection was used with a wavelength switch from 220 nm to 245 nm, typically at 1.75 minutes. The dissolving solvent for DD was 45:55 ACN:H_2_O, for NGI assessments and eLung the dissolving solvent was 10 µg/mL benzophenone in 80:20 DMSO:H_2_O for eLung assessments. The limit of quantification (LoQ) for NGI and eLung was 0.5 ng for FF and 1 ng for UMEC and VI. The reproducibility of the method for FF/UMEC/VI (100/62.5/25 µg) was 4.3 %, 3.1 % and 3.6 % relative standard deviation (RSD) respectively for each molecule, and 4.0 %, 2.6 % and 2.8 % RSD for dosing of 200/62.5/25 µg respectively. The HPLC method used was able to detect all three analytes via use of a single method with a wavelength switch capable of quantifying all components.

**Aerosol Particle Size Distribution by Next-Generation Impactor:** To achieve consistent assessment of the APSD, a composite of six doses are assessed from the device. A finite amount of powder is required to coat the metal throat before a consistent assessment of the particle size distribution can be achieved. Therefore, using composite doses decreases the variance which would be observed using a single dose where coating effect of the metal throat and lower sample concentration would affect the results.

**Supplementary Table 1. Mean delivered dose FF/UMEC/VI through the ELLIPTA DPI (percentage of nominal blister content).**

| **Flowrate (L/min)** | **Fluticasone furoate** | **Umeclidinium** | **Vilanterol** |
| --- | --- | --- | --- |
| **FF/UMEC/VI 100/62.5/25µg (Asthma and COPD)** | | | |
| 30 | 88.0 (83.3; 92.7) | 81.4 (76.3; 86.5) | 80.5 (75.8; 85.3) |
| 60 | 93.8 (89.1; 98.5) | 88.4 (83.2; 93.5) | 86.4 (81.7; 91.2) |
| 90 | 97.1 (92.4; 101.8) | 91.1 (86.0; 96.3) | 89.5 (84.8; 94.3) |
| **FF/UMEC/VI 200/62.5/25µg (Asthma)** | | | |
| 30 | 90.5 (89.9; 99.3) | 86.4 (81.3; 91.6) | 84.8 (80.1; 89.6) |
| 60 | 94.6 (98.9; 99.3) | 89.8 (84.7; 94.9) | 86.6 (81.9; 91.4) |
| 90 | 94.9 (90.2; 99.6) | 91.0 (85.9; 96.1) | 88.4 (83.6; 93.2) |

Data are presented as mean (95% confidence, 95% coverage tolerance intervals around the mean) values. At each flowrate, two doses from each of 10 inhalers were aerosolized into the test apparatus.
COPD, chronic obstructive pulmonary disease; DPI, dry powder inhaler; FF, fluticasone furoate; UMEC, umeclidinium; VI, vilanterol.

**Supplementary Table 2. Total delivered dose and fine particle fraction for FF/UMEC/VI through the ELLIPTA DPI as assessed through an NGI.***

| **Flowrate (L/min)** | **Fluticasone furoate** | | | **Umeclidinium** | | | **Vilanterol** | | |
| --- | --- | --- | --- | --- | --- | --- | --- | --- | --- |
|  | **Delivered dose (% nbc)** | **FPF (% nbc)** | **MMAD**  **(µm)** | **Delivered dose (% nbc)** | **FPF (% nbc)** | **MMAD**  **(µm)** | **Delivered dose (% nbc)** | **FPF (% nbc)** | **MMAD**  **(µm)** |
| **FF/UMEC/VI 100/62.5/25µg (Asthma and COPD)** | | | | | | | | | |
| 30 | 88.5 (84.9; 92.1) | 21.9 (20.2; 23.7) | 4.4 | 87.8 (84.5; 91.0) | 34.6 (31.3; 37.9) | 3.3 | 85.9 (82.4; 89.5) | 31.8 (28.3; 35.3) | 2.7 |
| 60 | 94.6 (91.0; 98.2) | 26.6 (24.9; 28.3) | 3.9 | 91.3 (88.1; 94.6) | 41.8 (38.5; 45.1) | 2.9 | 89.1 (85.6; 92.7) | 40.3 (36.7; 43.8) | 2.2 |
| 90 | 97.0 (93.4; 100.6) | 28.2 (26.5; 29.9) | 3.6 | 93.2 (90.0; 96.5) | 45.5 (42.2; 48.8) | 2.7 | 91.0 (87.5; 94.6) | 45.3 (41.7; 48.8) | 2.0 |
| **FF/UMEC/VI 200/62.5/25µg (Asthma)** | | | | | | | | | |
| 30 | 89.9 (86.3; 93.5) | 19.0 (17.3; 20.7) | 4.7 | 86.1 (82.8; 89.3) | 34.0 (30.7; 37.3) | 3.2 | 84.0 (80.4; 87.6) | 30.2 (26.7; 33.8) | 2.6 |
| 60 | 92.6 (88.9; 96.2) | 23.4 (21.7; 25.2) | 4.0 | 87.5 (84.3; 90.8) | 40.2 (36.9; 43.5) | 2.9 | 85.3 (81.7; 88.8) | 37.4 (33.8; 40.9) | 2.1 |
| 90 | 93.5 (89.9; 97.1) | 25.3 (23.6; 27.1) | 3.8 | 91.5 (88.3; 94.8) | 44.7 (41.4; 48.0) | 2.8 | 89.2 (85.6; 92.7) | 43.3 (39.8; 46.9) | 2.0 |

*Data are presented as mean (95% confidence, 95% coverage tolerance intervals around the mean) values. For each product strength and flowrate, four ELLIPTA DPIs were tested, with six doses actuated into the impactor from each device, resulting in a six-dose composite sample for analysis.
COPD, chronic obstructive pulmonary disease; DPI, dry powder inhaler; FF, fluticasone furoate; FPF, fine particle fraction; MMAD, median mass aerodynamic diameter; nbc, nominal blister content; NGI, Next Generation Impactor; UMEC, umeclidinium; VI, vilanterol.

**Supplementary Table 3. Total delivered dose and fine particle fraction for FF/UMEC/VI through the ELLIPTA DPI for five inhalation profiles assessed through the eLung.***

| **PIFR (L/min)** | **Inhaled volume (L)** | **Fluticasone furoate** | | | **Umeclidinium** | | | **Vilanterol** | | |
| --- | --- | --- | --- | --- | --- | --- | --- | --- | --- | --- |
|  |  | **Delivered dose (% nbc)** | **FPF (% nbc)** | **MMAD**  **(µm)** | **Delivered dose (% nbc)** | **FPF (% nbc)** | **MMAD**  **(µm)** | **Delivered dose (% nbc)** | **FPF (% nbc)** | **MMAD**  **(µm)** |
| **FF/UMEC/VI 100/62.5/25µg (COPD)** | | | | | | | | | | |
| 43.5 | 0.8 | 94.8 (88.4; 101.3) | 24.8 (21.7; 27.9) | 3.1 | 86.7 (81.9; 91.6) | 37.5 (33.7; 41.4) | 2.6 | 82.8 (77.6; 88.0) | 31.6 (27.4; 35.7) | 2.0 |
| 67.4 | 1.8 | 95.4 (89.0; 101.8) | 24.4 (21.3; 27.5) | 3.0 | 88.2 (83.3; 93.0) | 37.6 (33.7; 41.4) | 2.5 | 84.3 (79.2; 89.5) | 32.0 (27.9; 36.2) | 1.9 |
| 82.4 | 2.1 | 95.6 (89.2; 102.0) | 23.4 (20.3; 26.5) | 2.6 | 89.1 (84.3; 93.9) | 35.6 (31.7; 39.4) | 2.4 | 87.0 (81.8; 92.1) | 32.4 (28.2; 36.5) | 1.8 |
| 99.9 | 2.4 | 93.7 (87.3; 100.1) | 23.6 (20.5; 26.6) | 2.5 | 87.5 (82.7; 92.3) | 36.8 (32.9; 40.6) | 2.2 | 84.8 (79.7; 90.0) | 35.3 (31.2; 39.5) | 1.6 |
| 129.9 | 3.2 | 92.8 (86.4; 99.2) | 22.3 (19.2; 25.4) | 2.4 | 88.0 (83.1; 92.8) | 37.4 (33.6; 41.3) | 2.1 | 84.9 (79.7; 90.1) | 37.1 (33.0; 41.3) | 1.5 |
| **FF/UMEC/VI 200/62.5/25µg (Asthma)** | | | | | | | | | | |
| 67.4 | 1.8 | 90.2 (83.7; 96.6) | 24.2 (21.1; 27.3) | 2.9 | 88.9 (84.1; 93.7) | 34.5 (30.6; 38.3) | 2.6 | 85.3 (80.1; 90.5) | 32.3 (28.2; 36.5) | 2.1 |
| 82.4 | 2.1 | 90.0 (83.6; 96.4) | 24.2 (21.1; 27.3) | 2.6 | 88.8 (83.9; 93.6) | 34.7 (30.8; 38.5) | 2.4 | 85.1 (79.9; 90.2) | 33.8 (29.6; 37.9) | 2.0 |
| 99.9 | 2.4 | 90.1 (83.7; 96.5) | 24.1 (21.0; 27.1) | 2.4 | 90.2 (85.4; 95.0) | 37.7 (33.8; 41.5) | 2.2 | 86.6 (81.4; 91.8) | 39.8 (35.7; 43.9) | 1.7 |
| 113.1 | 3.3 | 90.8 (84.4; 97.2) | 22.5 (19.4; 25.5) | 2.1 | 89.8 (85.0; 94.6) | 36.2 (32.3; 40.1) | 1.9 | 86.3 (81.2; 91.5) | 41.6 (37.4; 45.7) | 1.5 |
| 129.9 | 3.2 | 90.8 (84.4; 97.2) | 23.1 (20.0; 26.2) | 2.2 | 92.3 (87.5; 97.1) | 33.2 (29.4; 37.1) | 2.2 | 88.2 (83.1; 93.4) | 34.4 (30.3; 38.5) | 1.8 |

*Data are presented as mean (95% confidence, 95% coverage tolerance intervals around the mean) values. For each product strength and flowrate, three ELLIPTA DPIs were tested, with six doses actuated into the impactor from each device, resulting in a six-dose composite sample for analysis.
COPD, chronic obstructive pulmonary disease; DPI, dry powder inhaler; FF, fluticasone furoate; FPF, fine particle fraction; MMAD, median mass aerodynamic diameter; nbc, nominal blister content; UMEC, umeclidinium; VI, vilanterol.
